# Glycemic and Insulinemic Responses of Vegetables and Beans Powders Supplemented Chapattis in Healthy Humans: A Randomized, Crossover Trial

**DOI:** 10.1155/2019/7425367

**Published:** 2019-10-13

**Authors:** Saeed Akhtar, Anam Layla, Piero Sestili, Tariq Ismail, Khurram Afzal, Albert A. Rizvanov, Muhammad Hassham Hassan Bin Asad

**Affiliations:** ^1^Institute of Food Science & Nutrition, Bahauddin Zakariya University, Multan, Pakistan; ^2^Department of Biomolecular Sciences, Sports Science and Health Department, University of Urbino ‘‘Carlo Bo” Via ‘‘The Maggetti”, 2661029 Urbino, PU, Italy; ^3^Institute of Fundamental Medicine and Biology, Department of Genetics, Kazan Federal University, Kazan, Russia; ^4^Department of Pharmacy, COMSATS University Islamabad, Abbottabad Campus, 22060, Islamabad, Pakistan

## Abstract

Vegetables and beans are nutrient-dense foods with innate potential to mediate diabetes in a variety of cultures. The present study aims at evaluating vegetables and beans for assessing their glycemic index and response in raising glucose levels in human model. Powdered formulations of vegetables and beans were designed to modulate glycemic response of carbohydrate-rich staples. A randomized, crossover trial was conducted in healthy young adults (*n* = 24) who were challenged with vegetable powder-supplemented chapatti (VPSC), bean powder-supplemented chapatti (BPSC) and all-purpose wheat flour chapatti (APFC) to evaluate their postprandial glucose (PPG) and postprandial insulin (PPI) responses. In comparison with APFC, feeding VPSC and BPSC to healthy volunteers anticipated significant reduction in PPG (44% reduction in incremental area under the curve (AUC) for VPSC and 46% reduction in incremental AUC for BPSC, *p* = 0.005). Likewise, significant reduction in PPI levels was observed for VPSC (59%, *p* = 0.012) and BPSC (47%, *p* = 0.002) compared to APFC-treated group. The study concludes wheat flour enrichment with vegetables and beans powder as a viable approach to develop cost effective and culturally acceptable low glycemic foods bearing acceptable sensory attributes.

## 1. Introduction

Diabetes is widely pervasive with 415 million diagnosed and 193 million undiagnosed cases. Moreover, about 12% share of global health expenditure is spent on the treatment of diabetes [1]. Asia has emerged as a major epicenter of diabetes epidemic, and the menace persistently prevails in most populous and middle-income countries of the region [2]. Poor control of diabetes evidently engenders risk of macrovascular and microvascular complications, resulting in the genesis of huge stress on the health-care system and socioeconomic status of the patients and caregivers. Reportedly, practicing a healthy dietary pattern with lifestyle modifications has come out as a potential strategy to avert diabetes [3].

Dietary habits that continually expose to post-meal hyperglycemia are believed to impair first-phase insulin secretion and decrease insulin sensitivity that might contribute an increased risk for insulin resistance and type 2 diabetes development [4]. Low glycemic index (GI) diets reduce postprandial glucose (PPG) by slowing digestion and the rate of nutrient influx from the gut and help to modulate insulin responses, thereby eliciting a beneficial effect in type 2 diabetes and other chronic maladies [5, 6]. Relatively increased consumption of carbohydrate-rich foods generally leads to heightened daily glycemic load, thus annunciating them as candidates of interest for reducing PPG and postprandial insulin (PPI) responses [7]. Wheat and rice are the most common carbohydrate-rich staples in the indigenous food system of South Asia and are equally popular amongst Asian immigrant communities worldwide [8]. Glycemic indices of cereal based formulations like wheat and millet had been reported relatively lower than white rice recipes [9]. However, manipulation in comparatively high GI white rice recipes is practiced by replacing one serving with that of a low GI cereal to suppress PPG load [10].

Chickpea and red kidney beans are the low GI foods that hold substantial amounts of soluble fibers like galactomannan and resistance starch [11]. Delayed gastric emptying is referred as one amongst the possible mechanisms for improved metabolic control in diabetic patients [12]. Dietary fibers including those derived from beans increase the response of cholecystokinin – a hormone responsible for delayed gastric emptying and provoking satiety for food [13, 14]. Moreover, earlier researches also advocate fiber-anticipated viscosity and anti-nutrient-linked reduction in the rate of food digestion as possible reasons for delayed gastric emptying [15, 16].

Consumption of vegetables has been shown to evoke positive effect amongst diabetics by improving PPG excursions and insulin sensitivity [17]. Roots of turnip and radish are good sources of soluble fibers and minerals, especially magnesium and zinc, which are necessary cofactors for enzymes that improve glucose metabolism and insulin signaling pathways. Leafy vegetables such as mustard and cabbage have been explored for hypoglycemic and insulin sensitizing potential and suggested as adjuvants to oral hypoglycemic drugs [18, 19]. Likewise, trace amounts of added cumin seeds (*Nigella sativa* L.) as part of its essential oil are physiologically meaningful in suppressing PPG [20]. These excelling features of beans and vegetables are of tremendous interest for researchers and health professionals to design low glycemic response cereal-based food products. The research in question was conducted to develop vegetable powder-enriched and beans powder-enriched wheat flour premixes for preparing organoleptically acceptable chapattis, and to evaluate PPG and PPI responses of the modified recipes in healthy human subjects.

## 2. Materials and Methods

### 2.1. Raw Materials

Commercially milled wheat flour, black cumin, red kidney beans, and chickpeas were sourced in one lot from the local supermarket of District Multan, Pakistan. Vegetables such as mustard, cabbage, turnip, and radish were procured from agro farm fields of city vicinity during their peak seasons. Analytical grade chemicals and reagents were used to perform biochemical analysis of the powdered vegetables and beans were procured from Merck (Darmstadt, Germany) and Sigma Chemical Co., Ltd. (St. Louise, MO) unless otherwise mentioned.

### 2.2. Production of Various Types of Composite Flour

Four different types of flour blends were prepared to elucidate the best organoleptic response using composite flour technology. All-purpose wheat flour being the base material was replaced at different proportions with bean and vegetable powder (VP) (Table 1).

### 2.3. Preparation of Chapattis

The newly developed flour blends along with control flour were used to make chapattis. Flour mixes were kneaded till soft consistency by the addition of water. Finally, the dough was developed and allowed for proofing duration of 30 minutes. The dough balls were rolled out with wooden rolling pin, and dough sheets measuring 18 cm were developed. Flatbreads having thickness of 3 mm each were baked on a hotplate at 210°C for 150 s [21].

### 2.4. Organoleptic Evaluation

Chapattis were organoleptically evaluated to select the best combination on the basis of appearance, color, taste, texture, folding ability, and acceptability on 9-point hedonic scale [21, 22]. Organoleptically acceptable best quality baked chapattis made of composite flours were further used for glycemic response assessment. Sensory properties of the product were carried out by sensory experts' panel from the Institute of Food Science & Nutrition, Bahauddin Zakariya University, Multan, Pakistan, with good product discriminative ability for different sensory attributes. The product was served to the experts under white light in the absence of food/chemical odor and unnecessary sound in the sensory analysis laboratory. Distilled water and crackers were used to clean the mouth between samples analysis. The panelists were provided with questionnaires to record their observations on 9-point hedonic scale.

### 2.5. Nutritional Profiling

Nutritional composition, including protein (920.87), fiber (920.86) and lipids (923.05) contents of chapattis with the best organoleptic response, was assessed by the methods laid down in AOAC manual of food analysis by Latimer [23].

### 2.6. Determination of Available Carbohydrates

Available carbohydrate contents of chapattis (dry weight basis) were measured by following phenol-sulphuric acid method [24]. One hundred milligrams of a homogeneous sample of dried chapattis powder was hydrolyzed in boiling water with 5 ml 2.5N HCl for 3 hr and neutralization was performed with sodium carbonate crystals. Boiling tubes carrying samples were cooled to 25°C. Samples were centrifuged, and 5 ml of H_2_SO_4 _was rapidly added in supernatant (2 ml); subsequently, 1 ml of 5% aqueous phenol was added to tube contents. The samples were vortexed (30 s) and kept at room temperature for 20 min. The intensity of color resulting so was measured against blank at 490 nm using a spectrophotometer (UV–Vis 3000, ORI, Germany). All chapatti samples were tested in three replicates and carbohydrate concentrations were determined using glucose standard solutions of known concentration.

### 2.7. Glycemic Indexing and Insulin Response Assessment of BPSC and VPSC Chapattis

#### 2.7.1. Participants of the Study

Normoglycemic healthy young adults were recruited from staff/students of Bahauddin Zakariya University, Multan, Pakistan. The study was approved by a bioethics committee of Bahauddin Zakariya University, Multan, Pakistan (Reg. No. 04-18/2018), and was executed taking into consideration the ethical principles mentioned in the Declaration of Helsinki. Eligible participants were normoglycemic healthy individuals entailing age limit (18–30 years), body mass index (18.5–24.9 kg/m^2^)and fasting glucose levels (<6 mmol/L). The exclusion criteria were built on smoking, excessive alcohol consumption (>50 g/day), body weight change >5% in the past six months, pregnancy or lactation, any type of chronic metabolic disease, regular use of supplements/medications, for example, birth control pills, anti-asthmatic, diuretics that might interfere with glucose and lipid metabolism, or those unwilling to follow the study protocol. Of total twenty-seven screened, selected subjects, three refused to participate due to unavailability to attend all test sessions. Finally, twenty-four enrolled subjects provided written informed consent and completed the study. Participants were physically examined, and base line demographic data were assessed before GI testing.

#### 2.7.2. Reference Food/Test Meal

The reference beverage was prepared by dissolving 55 g dextrose monohydrate (Glaxose-D glucose powder, Unilever Pakistan Food Ltd., Karachi, Pakistan) in 250 ml water and was dispensed to the subjects during test sessions.

Selected BPSC and VPSC were freshly prepared at serving time in the metabolic kitchen of the research center and were served with fried egg (54 g wt., 15.4% lipids, 13.3% protein, 0.6% carbohydrates) cooked in sunflower oil. Meal palatability was increased by serving 250 ml water to the participants along with the meal. Available carbohydrate contents were used to calculate portion size of the test foods.

#### 2.7.3. Experimental Protocol

Subjects were challenged with three experimental diets, that is, (1) APFC (control), (2) BPSC, and (3) VPSC in random order on non-consecutive week days. Assessment was conducted in four non-consecutive week days over a period of 6–8 weeks during July 2018–August 2018, and an interval of two weeks was set between visits. The proportions of each diet were standardized to yield 50 g available carbohydrate content. Glucose beverage equivalent to deliver 50 g available carbohydrates was used as a standard for GI determination. The experimental procedure used to measure GI was adopted by FAO/WHO recommended method [25]. The study subjects were individually informed to avoid vigorous physical activity and smoking. Brief behavioral questionnaire and 24-hour recall were used to ensure balanced evening meal consumption by the participants. Subjects were asked to visit research center early morning with ~12 hr fasting, but were allowed to drink water *ad lib* during overnight fast. Fasted subjects were provided with standard glucose beverage or freshly prepared chapattis. All subjects were advised to finish their test meals within 15 min. Blood sampling was performed during 2 hours post-test meal consumption for PPG and PPI analysis.

#### 2.7.4. Blood Glucose and Insulin Measurements

Capillary blood sampling was performed in the fasted subjects at −5 and 0 min (two baseline measurements). Postprandial blood sampling was performed at 15, 30, 45, 60, 90 and 120 min from the baseline sampling. Blood glucose concentrations were assessed using a lancet device manufactured by Accu-Chek Performa, Roche Diabetes Care GmbH, Germany. The device was calibrated every day prior to testing, using control solution and the blood chemistry analyzer (Biosystem BTS-350, Spain). Insulin response was assessed by drawing 2 ml postprandial venous blood samples in serum separator vacutainers at 0, 30, 60 and 120 min for the estimation of insulin response. Blood samples were centrifuged for 5 min at 3000 rpm and 25°C. Serum samples were collected in the 1.5 ml labelled Eppendorf tubes and stored in ultralow temperature freezer (MDF-U33V-PB, Japan) at −70°C. Serum insulin concentrations were measured on microtiter plate reader using the sandwich ELISA technique with immunoassay kit (Human insulin, Chemux BioScience, South San Francisco) with standard range of 5–200 U/mL.

### 2.8. Data Analysis

Physicochemical analysis data of products were statistically analyzed by analysis of variance (ANOVA) technique using computerized program Statistix 8.1 (Tallahassee, FL). Trapezium rule without using values below the baseline was used to perform time point differences analysis and incremental area under the curve (iAUC) calculations for blood glucose and insulin response assessment [25]. Differences amongst the means were determined by post-hoc analysis and confidence interval was set at 5% (*p* < 0.05). Graphs were designed using GraphPad Prism 7.0 (CA, USA) [26].

## 3. Results

### 3.1. Organoleptic Evaluation of VPSC and BPSC

Data presented in Table 2 showed that the addition of VP antagonistically influenced color and appearance (*p* < 0.05) that render a typical out look to the product under sensory evaluation. The addition of VP (20% & 30%) and BP (50%) slightly deteriorated the texture characteristics of VPSC and BPSC nevertheless chapattis remained acceptable to judges. Supplementation of BP at 50% and 25% and VP at 20% in APF to make VPSC and BPSC did not negatively affect the taste of the finished product (Table 2).

### 3.2. Nutritional Profiling of VPSC and BPSC

Nutritional composition of VPSC and BPSC balanced out to deliver 50 g of available carbohydrates is stated in Table 3. Nonsignificant (*p* < 0.05) difference was observed in carbohydrate contents of VPSC and BPSC, but supplementation of APF with VP and BP evidently enhanced nutrient profile of chapattis when compared with control. Likewise, the addition of BP (25%) to APF exhibited slightly higher crude fat level in BPSC (1.14 g), while the addition of VP (20%) failed to change (*p* > 0.05) total fat in VPSC, as compared to the chapattis made by APF only. Interestingly, the addition of cruciferous vegetable and bean powder, both being good sources of proteins, significantly (*p* < 0.05) improved protein contents of VPSC and BPSC by 41% and 43%, respectively, when compared with APFC.

### 3.3. Glycemic Indexing and Glycemic Load Assessment

Baseline clinical and anthropometric characteristics of the subjects separated by sex have been presented in Table 4. The mean fasting capillary glucose of subjects was 5.2 mmol/L. Postprandial glucose responses of experimental foods using iAUC and their GI values are summarized in Table 5. In comparison with the APFC bearing highest GI, that is, 82, lowest GI values were recorded for chapattis made of BPSC (44), and VPSC (46), that represented 46% and 44% decrease in GI values of BPSC and VPSC, respectively.

### 3.4. Postprandial Glycemic and Insulinemic Responses

**Figure 1:**
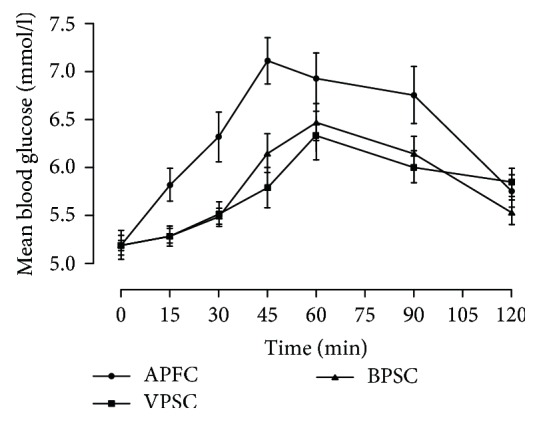
Glycemic response to low and high glycemic index (GI) chapattis.

**Figure 2:**
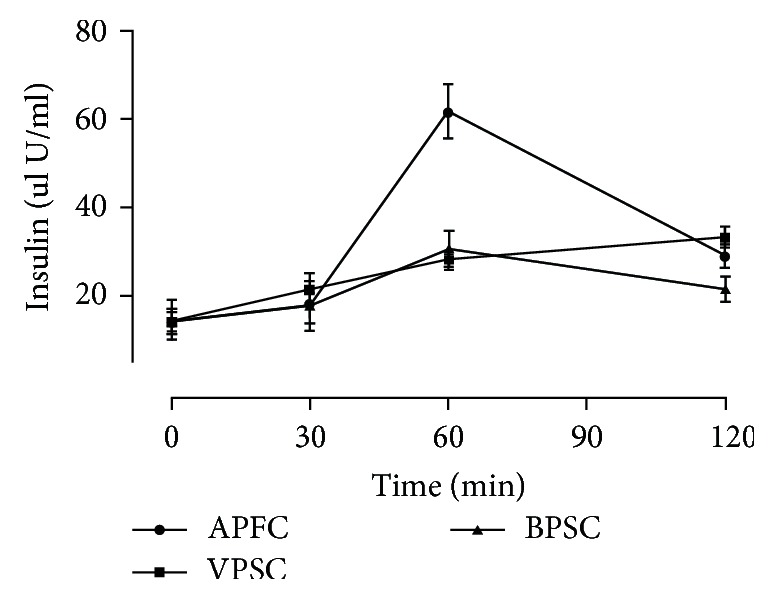
Insulin response to high- and low-glycemic index chapattis.

The postprandial glycemic response curves comparing the high GI (APFC) and low GI (VPSC and BPSC) food products are illustrated in Figure 1. The low GI-chapattis flattered glycemic response and shifted the response curve downwards, as compared to APFC. Time point differences in postprandial blood glucose concentrations were statistically lower at 15 min (*p* = 0.022), 30 min (*p* = 0.018) and 45 min (*p* = 0.0018) for VPSC. Significant differences in the postprandial glucose concentration were also observed for BPSC treated group at 15 min (*p* = 0.017), 30 min (*p* = 0.012) and 45 min (*p* = 0.011). The postprandial glucose response to BPSC at 120 min tended towards lower side than APFC and VPSC. Significant reduction in iAUC_0–120 min_ values of blood glucose was observed for VPSC (49%) and BPSC (44%) in comparison with the normal control (*p* = 0.005). Sixty minutes post ingestion of VPSC and BPSC also revealed significantly (*p* = 0.0003) lower values of insulin as compared to APFC (Figure 2). Amplitude of postprandial insulin responses was significantly shortened and lowered for VPSC (*p* = 0.012) and BPSC (*p* = 0.002) as compared to the APFC.

## 4. Discussion

The data presented above confirmed supplementing vegetable and bean powder to the refined carbohydrate staples may improve short-term glycemia and insulinemia; however, could not be generalized to the individuals with type 2 diabetes. Reduction in taste of VPSC at 30% supplementation is predominately associated with mustard leaves and the study validates findings of Chin & Lindsay [27] who reported isothiocyanates producing sulfurous aroma and pungent flavor from shredded and cooked brassica tissues. Results of the present study for various sensory traits of VPSC and BPSC were consistent with those of folding ability and overall palatability: the combination *T*_2_ and *T*_3_ ranked the lowest in the overall acceptability of the baked chapattis. This finding is consistent with the analogous observation that supplementing cereals with unconventional food crops beyond 25% reduces consumers' acceptability index [28, 29]. Based on the above-mentioned sensorial observations, VPSC and BPSC prepared from the flour blends *T*_1 _(80% APF + 20% VP) and *T*_4_ (75% APF + 25% BP) were used in nutritional profiling of the products and their glycemic response assessment. Irrespective of matchable carbohydrate profile, higher protein and fat contents in VPSC and BPSC contributed 56–70 calories per serving higher than APFC. In comparison with control, that is, APFC, 3.6–3.9 g higher protein and 1.7–3.1 g higher fiber contents per serving were observed in VPSC and BPSC that may contribute significant differences in glycemic and insulinemic performance. In analogy with our results (Table 3), better nutritional profiles were reported for composite baked -roll cakes produced from soybean and vegetable flour instead of wheat flour [30]. These findings further strengthened the notion that new formulations (VPSC & BPSC) may deliver more calories from non-carbohydrate sources that might be beneficial for individuals with insulin resistance.

Supplementation of VP (20%) and BP (25%) in APF significantly (*p* < 0.0024) reduced mean iAUC of the tested products. The assigned GI values for newly developed recipes (VPSC and BPSC) non-significantly (*p* ≥ 0.05) differed from each other suggesting both formulations to be equally beneficial in reducing postprandial blood glucose spikes. Several groups of investigators reported low (≤55) to high (≥70) GI scoring to wheat flour chapattis [31, 32] that could be associated with myriad of factors including varied fiber, starch and gluten contents, method of processing or cooking, presence of anti-nutrients, slowly digestible and resistant starch which were corollary to influence GI values [33]. Higher GI values, that is, ≥70 have been reported for white flour bread, while whole wheat flour and fiber supplementation in white wheat flour have been cited with low GI values, that is, ~54% [34]. The results of this study are in agreement to the findings of Radhika et al. [31] and Gopalpura et al. [35] who reported wheat flour formulations mixed with legumes (Bengal grams flour) and soluble viscous fibers (psyllium and fenugreek powder) to demonstrate their potential in lowering GI values of chapattis. The outcomes of the current study point to the potential of VPSC and BPSC to lower GI. Hence, due to their lower glycemic response, both VPSC and BPSC might represent a valuable tool in diabetes management, more particularly in regions reported with consumption of high glycemic load-bearing staples.

Our study clearly shows that VP and BP mixes are viable formulations as compared to standard APFC to lower PPG and PPI responses in the context of the Asian population, where escalating prevalence of type 2 diabetes appears to be a serious threat and public health issue. Our findings are corroborated by the upshots of foregoing exploratory studies where combinations of bean powder and fiber mixes with wheat flour decreased PPG (≥30% reduction in iAUC_0–20 min_) and PPI (≥28% reduction in tAUC_0–120 min_) as compared to commercial wheat flour-based chapattis [36, 37]. Another study by Johnson et al. [38] reported 35% chickpea flour incorporation to wheat flour significantly decreased glycemic response of the products. Contrarily to the use of mixed grains formulations in hyperglycemia management, single chickpea-based meal has also been reported to lower both the glucose and insulin amount (55% reduction in PPG at 30 and 60 min while PPI at 120 min time point) [39].

The results of this study seem to be novel in that the developed formulations using VP and BP mixed with APF for making chapattis are significantly effective in reducing PPG and PPI levels. The present findings on PPG and PPI responses for VP mix chapatti served with fried egg could not be compared with earlier studies wherein vegetable curries served with wheat flour chapattis significantly reduced PPG and PPI [40, 41]. We developed and focused on combinations of various vegetables and beans in the form of powder mixed with wheat flour, while retrospective studies focused on vegetables dispensed as curries. Likewise, Imai et al. [17] suggested the inclusion of vegetables before eating carbohydrates meal considerably reduce glucose excursions and PPG responses (≥39% reduction in iAUC_0–3 hour_) in type 2 diabetes patients. The therapeutic effects of vegetables on lowering PPG and PPI levels are probably due to high fibers and lente carbohydrates which exhibit viscous and gel-forming properties. Such a composition may lead to delayed rate of gastric emptying and nutrients absorption and finally result in lower PPG response that requires less insulin for subsequent metabolic disposal [19, 42]. To this regard, it may be presumed that fibers and bioactive compounds from vegetable powder through the modification of gastric conditions for hydrolytic enzymes activity result in slower release of nutrients thereby lowering PPG and PPI responses. Furthermore, cruciferous vegetables—largely included in our VP—are a uniquely rich source of isothiocyanates that are known to help in blood glucose control and beneficially influence insulin sensitivity [43].

The ability of chickpeas and red kidney beans to mitigate high glycemic response has been reported in many studies where the impact of dried beans on PPG and PPI was investigated in both healthy and type 2 diabetes subjects consuming high glycemic carbohydrates [44–46]. The postprandial glucose lowering potential of red kidney beans was attributed to viscous soluble fibers—mainly galactomannan and amylose rich starch granules—which might have stayed intact during simulated digestion [46]. Likewise, chickpea flour delivers highest amount of slow release starches amongst pulses that reduce post meal gastric emptying rate and glucose absorption to modulate PPG and PPI responses [47].

Findings of this recent study and literature as discussed above confirmed that partial replacement of wheat flour with dehydrated beans and vegetables is a likely approach for lowering PPG and PPI responses due to their higher contents of fiber as compared to the counterpart. Future research may identify the role of resistant and slow release starches from beans and vegetables powder to influence PPG and PPI responses in supplemented foods like chapattis. The study suggests explorative research to evaluate metabolic effects of vegetables and beans powder mix consumption in peoples with type 2 diabetes, and to investigate ghrelin, incretin and glucagon responses towards these kinds of flour formulations to better understand overall nature of their physiological responses.

## Figures and Tables

**Table 1:** Ingredient-based composition of treatment models.

Ingredient (g)	*T*_0_	*T*_1_	*T*_2_	*T*_3_	*T*_4_
Wheat flour	100	80	70	49.5	74.5
Mustard powder	0	3	6	0	0
Cabbage powder	0	7	10	0	0
Turnip powder	0	5	7	0	0
Radish powder	0	5	7	0	0
Kidney beans flour	0	0	0	20	10
Gram flour	0	0	0	30	15
Powdered black cumin	0	0	0	0.5	0.5

*T*_0_ = Control; *T*_1_ and *T*_2_ = Vegetable powder-supplemented treatments; *T*_3_ and *T*_4_ = Bean powder-supplemented treatments. ∗Glycemic response assessment was performed only for the treatments with the best organoleptic responses.

**Table 2:** Sensory evaluation of APFC, VPSC and BPSC prepared with different combinations.

Product Type	Appearance	Color	Texture	Taste	Folding ability	Overall palatability
*T*_0_	7.80 ± 0.27^a^	7.30 ± 0.24^a^	7.50 ± 0.19^a^	7.20 ± 0.3^a^	7.58 ± 0.19^a^	7.49 ± 0.27^a^
*T*_1_	6.85 ± 0.28^bc^	6.63 ± 0.24^b^	6.53 ± 0.19^bc^	7.02 ± 0.31^a^	7.04 ± 0.19^ab^	6.86 ± 0.28^ab^
*T*_2_	6.19 ± 0.33^c^	6.50 ± 0.28^b^	5.84 ± 0.28^c^	5.47 ± 0.37^b^	6.64 ± 0.25^bc^	6.13 ± 0.36^c^
*T*_3_	7.00 ± 0.23^b^	7.36 ± 0.18^a^	6.17 ± 0.35^c^	6.56 ± 0.31^a^	6.15 ± 0.34^c^	6.69 ± 0.26^bc^
*T*_4_	7.30 ± 0.19^ab^	7.50 ± 0.21^a^	7.15 ± 0.27^ab^	7.26 ± 0.24^a^	7.23 ± 0.26^ab^	7.33 ± 0.13^ab^

*T*_0_ = 100% PF: *T*_1_ = 80% PF + 20% VP: *T*_2_ = 70% PF + 30% VP: *T*_3_ = 50% PF + 50% BP: *T*_4_ = 75% PF + 25% BP. ∗ Means sharing similar lettering in a column is nonsignificantly different at *p* ≥ .05, Mean ± SD.

**Table 3:** Nutritional composition of APFC, VPSC and BPSC selected on the basis of sensory responses—50 g ACB.

Nutrients (per serving)	*T*_0_	*T*_1_	*T*_4_
Energy (kJ)	959.1	1015.3	1029.9
Total fat (g)	0.81	0.90	1.14
Protein (g)	5.05	8.62	8.92
ACB (g)	50	50	50
TDF (g)	3.60	6.72	5.33
Serving size (g)	91.2	96.10	93.4

ACB = Available carbohydrates basis; TDF =  total dietary fiber: *T*_0_ = 100% PF: *T*_1_ = 80% PF + 20% VP: *T*_4_ = 75% PF + 25% BP. Mean ±  SD.

**Table 4:** Gender-based demographic characteristics of the subjects administered APFC, VPSC and BPSC.

Subjects characteristics	Male (*n* = 12) Mean ± SD	Female (*n* = 12) Mean ± SD
Age (years)	21.1 ± 1.2	23.8 ± 2.6
Height (m)	1.7 ± 0.04	1.6 ± 0.07
Weight (kg)	66.7 ± 4.5	56.3 ± 7.7
BMI (kg/m^2^)	22.5 ± 1.7	21.0 ± 1.7
WC (in)	33.7 ± 1.1	32.3 ± 1.3
FBG (mmol/l)	5.3 ± 0.3	5.2 ± 0.3

WC = waist circumference: FBG = fasting blood glucose; Mean ± SD.

**Table 5:** Incremental area under curve (iAUC) and glycemic index of tested vegetable powder and beans powder products.

Group	Mean iAUC ± SEM	GI ± SEM	Glycemic ranking
APFC	150 ± 16^a^	82 ± 16^a^	High
VPSC	75 ± 14^b^	46 ± 14^b^	Low
BPSC	84 ± 11^b^	44 ± 11^b^	Low

∗ Means sharing similar lettering in a column is nonsignificantly different at *p* ≥ .05, Mean ± SE.

## Data Availability

The data used to support the findings of this study are available from the corresponding upon request.
